# A neuraminidase activity-based microneutralization assay for evaluating antibody responses to influenza H5 and H7 vaccines

**DOI:** 10.1371/journal.pone.0207431

**Published:** 2018-11-15

**Authors:** Hui Zhao, Kangwei Xu, Zheng Jiang, Ming Shao, Shuzhen Liu, Xuguang Li, Junzhi Wang, Changgui Li

**Affiliations:** 1 Division of Viral Vaccine III, National Institutes for Food and Drug Control and WHO Collaborating Center for Standardization and Evaluation of Biologicals, Beijing, P.R. China; 2 Center for Biologics Evaluation, Ottawa, Biologics and Genetic Therapies Directorate, Health Canada and WHO Collaborating Center for Standardization and Evaluation, Ottawa, Canada; 3 Department of Biochemistry, Microbiology and Immunology, University of Ottawa, Ottawa, ON, Canada; University of South Dakota, UNITED STATES

## Abstract

Outbreaks of the highly pathogenic avian influenza H5N1 and H7N9 viruses have spurred an unprecedented research effort to develop antivirals and vaccines against influenza. Standardized methods for vaccine evaluation are critical for facilitating vaccine development. Compared with hemagglutination inhibition assays, mounting evidence suggest that microneutralization tests (MNTs) is a better choice for the evaluation of candidate pandemic influenza vaccines because they measure neutralizing antibody activity in cell cultures and are more sensitive in detecting H5 and H7. Here, we report a MNT measuring neuraminidase activity as the read-out (NA-MNT) for quantitative analysis of neutralizing antibodies against avian influenza viruses. Compared to the conventional microneutralization assay (ELISA-MNT), the NA-MNT is faster with lower intra- and inter-assay variations, while no difference in geometric mean titers was found between these two assays for the evaluation of H5N1 and H7N9 vaccines. These results suggest that NA-MNT is a reliable and high throughput method which could facilitate the development of candidate pandemic influenza vaccine.

## Introduction

Newly emerged avian influenza A viruses have a significantly negative impact on public health. Specifically, the highly pathogenic avian influenza H5N1 virus has infected 860 humans, with a mortality rate of 52%, according to the World Health Organization [1], whereas H7N9, first emerged in 2013, has infected over 1500 humans and caused 612 deaths [2]. Effective vaccines against these viruses in humans are urgently needed. As an important part of vaccine development, standard and reliable methods with high-throughput capacity are needed to evaluate the immune response elicited by influenza vaccines.

Several candidate assays, including neutralization and hemagglutination inhibition (HI) assays, have been used to assess the efficacy of the influenza vaccines. The microneutralization test (MNT) has proven to be useful in evaluating the immunogenicity of pandemic influenza H5N1 or H7N9 vaccines [3–6], as well as determining the prevalence of H5N1 in human populations that have had contact with infected birds, given that it measures the neutralizing activities of antibodies with greater sensitivity than the traditional HI assays [3, 4, 7]. The MNTs have a similar neutralization step, in which sensitive cells are inoculated with a mixture of viruses and serum, but have different final steps as read out including microscopically observing cytopathic effects (CPEs), assessing virus hemagglutination with red blood cells, or detecting viral proteins by ELISA. Using the CPE method, some influenza viruses induce uncharacteristic CPEs, which makes judgment of the results subjective and dependent on the experience of the observer. Although ELISA-MNT is more sensitive than HI as it quantifies viral nucleoproteins by ELISA, it is a relatively long procedure with multiple steps, making it a challenge for assay standardization. Indeed, considerable variations have been observed in inter-laboratory comparison studies [6, 8].

In this study, we describe an MNT that measures neuraminidase (NA) activity as the readout (NA-MNT). Only lysates from cells infected with virus and NA substrates are needed. This simple method was used here to measure antibody titers induced by pandemic influenza vaccines in comparison with results generated by both HA assay and ELISA-MNT.

## Materials and methods

### Cells and viral strains

MDCK (Madin-Darby canine kidney) cells were cultured in Dulbecco’s modified essential medium (DMEM) containing 1% penicillin/streptomycin (Gibco Life Technologies, Grand Island, NY, USA), 10% fetal calf serum (Hyclone South logan, UT, USA) and 1% L-glutamine and maintained in a 5% CO2 incubator at 37°C. The viruses tested were vaccine strains obtained from The National Institute for Biological Standards and Control (NIBSC; Potters Barr, UK), including A/Vietnam/1194/2004 NIBRG-14 (H5N1) and A/Anhui/01/2013 NIBRG-268 (H7N9). All viruses were grown in 10-day-old embryonated chicken eggs for 48–96 h at 35°C. Cellular debris from harvested allantoic fluids was removed by centrifugation at 3000 rpm for 10 min, with the harvested viral aliquots being stored at −70°C. The infectious titers of the viral stocks were determined as described previously [9]. The 50% tissue culture infective dose (TCID50) was calculated using Reed–Muench formula.

### NA assays

NA assays were performed as described previously [10] using the substrate 2-O-(4-methylumbelliferyl)-a-D-N-acetylneuraminic acid (MU-NANA) (Sigma-Aldrich, St Louis, MO, USA). Cleavage of MU-NANA by NA releases fluorescent methylumbelliferone which is subsequently quantified using a fluorescence plate reader.

### Human serum samples

A panel of serum samples was collected from 40 volunteers who received two doses of inactivated H5N1 influenza vaccine (A/Vietnam/1194/2004 NIBRG-14, 15 μg HA/dose), kindly provided by Wuhan Institute of Biological Products Co., Ltd, Wuhan, China. The trial was approved by the Ethical Committee of Jiangsu Provincial Center for Disease Control and Prevention and registered at chinadrugtrials.gov.cn (ID: CTR20131644). Serum samples were also collected from human subjects vaccinated with H7N9 influenza vaccine (A/Anhui/01/ 2013 NIBRG-268, 30 μg HA/dose), kindly provided by Hualan Biological Engineering Inc., Xinxiang, China. Individuals received two vaccinations (on days 0 and 21), while serum samples were collected on days 0, 21 and 42. The study was approved by the Ethical Committee of Henan Provincial Center for Disease Control and Prevention (chinadrugtrials.gov.cn; ID: CTR20160688). All samples were obtained from participants after they signed a written informed consent document.

### HI assays

HI assays were performed according to the standard protocol [11]. Briefly, serum samples were first treated with a receptor-destroying enzyme (cholera filtrate, Sigma-Aldrich) and adsorbed with chicken red blood cells to remove non-specific inhibitors of agglutination. 25 μl of the treated serum were then prepared as two-fold serial dilutions with 0.85% sodium chloride and incubated for 45 min at room temperature with the same volume of the virus preparations containing 4 HA units. The mixture was then further incubated with 25 μl 1% chicken red blood cells for 45 min at room temperature. Finally, the HI titer was determined as the reciprocal value of the last dilution that completely inhibited hemagglutination.

### ELISA-MNT

The ELISA-MNT was performed as described previously [9]. Briefly, five-fold dilutions were made for all serum samples with DMEM and heat-inactivated at 56°C for 30 min. They were then serially diluted by two-fold with DMEM containing 1% bovine serum albumin (BSA) and incubated with 50 μl influenza virus (100 TCID_50_) for 1 h at 37°C. The viral mixture was then added to MDCK cells and incubated for 20 h. Afterwards, the cells were fixed with 80% acetone for 10 min and blocked with 2% BSA for 1 h at room temperature. Following incubation with mouse monoclonal anti-NP (anti-Nucleoprotein) antibodies (Beijing Wantai Biological Pharmacy Enterprise Co., Ltd) for 1 h, the samples were washed three times with PBS containing 0.5% Tween 20 and then incubated with horseradish peroxidase-conjugated goat anti-mouse IgG antibodies for 1 h at 25°C. The plates were then washed six times, followed by addition of 100 μl 3,3´,5,5´-tetramethylbenzidine substrate (Kirkegaard and Perry Laboratories Inc., Gaithersburg, MD, USA) to each well. The reactions were terminated by addition of 0.4 M H_2_SO_4_. The plates were read at 450/620 nm using a microplate reader (Multiskan Ascent, Thermo Scientific, Waltham, MA). The neutralization titer was defined as the dilution of serum inhibiting 50% virus growth. The average absorbance values of virus-infected cells (VCs) and control cells (CCs) were used to calculate the 50% specific signal (X) by the following equation: X = [(average absorbance of VC wells)–(average absorbance of CC wells)] / 2 + (average absorbance of CC wells) [9]. All values less than X were considered positive for neutralization. The endpoint titer was recorded as the reciprocal of the highest dilution with an absorbance less than X.

### NA-MNT

Pre-treated serum (50 μl) was serially diluted by two-fold and then mixed with an equal volume of 100 TCID_50_ virus prepared in 96-well plates, followed by incubation for 1 h at 37°C with 0.5 μg/ml fexog-enousL- 1-tosylamide-2- phenylethyl chloromethylketone (TPCK)-treated trypsin (Sigma Immunochemical Co., St. Louis, Mo.). MDCK cells (4 × 10^4^/well) were then incubated with the serum and virus mixture for 20 h at 35°C in a 5% CO_2_ humidified incubator. Next, 50 μl of the culture supernatant were transferred to a new plate for substrate development. After the remaining supernatant was aspirated and discarded, 50 μl PBS containing 0.5% Triton-X were added to the cell layer and incubated for 10 min at room temperature. Afterwards, 50 μl 2-(n-morpholino) ethanesulfonic acid containing 20 μM 4-MU-NANA and 4 mM CaCl_2_ were added to the cell lysates, and the samples were incubated for an additional 1 h at 37°C before the reactions were stopped using 100 μl 0.2 M Na_2_CO_3_. Finally, relative fluorescence units (RFU) were quantified using a plate reader (Fluoroskan Ascent FL, Thermo Scientific) at excitation and emission wavelengths of 355 nm and 460 nm, respectively. VCs or CCs were included in each plate. The 50% signal inhibition (X) was calculated as follows: X = [(mean RFU of VC wells)–(mean RFU of CC wells)] / 2 + (mean RFU of CC wells). The neutralization titer endpoint was recorded as the reciprocal of the highest serum dilution that inhibited 50% viral replication.

### Statistical analysis

For Geometric mean titer (GMT) calculations, antibody titers (<10) were assigned a titer of 5. The HI and neutralization titers were converted to logarithmic values. The non-parametric Wilcoxon signed rank test was used to compare the data obtained from the supernatants and lysates. P values <0.05 were deemed as statistically significant. The correlations among the three methods were assessed by linear regression analysis using GraphPad Prism (GraphPad, San Diego, CA, USA).

## Results

### Optimization of the NA-MNT

**Fig 1:**
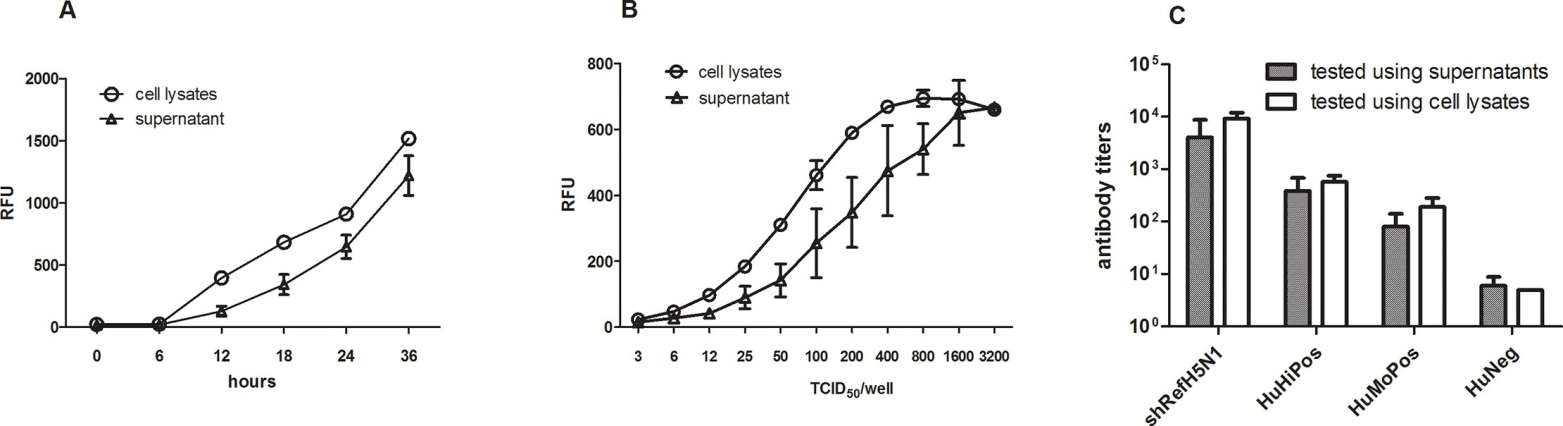
Comparison of NA activity between cell lysates and supernatants. (A) NA activity in cell lysates and supernatants at different time points following infection. Cells were infected with NIBRG-14 (H5N1) virus at a multiplicity of infection of 0.001. Each data point represents the mean of three separate experiments. (B) NA activity in cell lysates and supernatants with different viral inocula. MDCK cells were infected with different dilutions of NIBRG-14, with NA activity measured in either the cell culture supernatant or cell lysates 20 h after inoculation. The total NA activity in the supernatants was calculated and compared with the activity in the cell lysates. (C) The neutralizing antibody titers of serum samples measured by the NA-MNT. The neutralizing antibody titer of NIBSC reference sheep antisera (shRef H5N1), high (huHiPos) and moderate (HuMoPos) seropositive samples, and negative (huNeg) human sera were determined by NA-MNT using either cell supernatants or lysates. Each test was repeated five times, with the CVs calculated accordingly. Note: HuHiPos, HuMoPos, and huNeg samples were obtained from a clinical study of the H5N1 candidate vaccine.

In this study, the NA-MNT was developed using 4-MU-NANA as a substrate. Specifically, we performed assays and calculated the standard deviations (SDs) using cell lysate or supernatant samples containing virus. To this end, cells were first infected with virus at a multiplicity of infection of 0.001 and incubated for up to 36 h. As shown in Fig 1A, NA was detected 6 h after incubation, with significantly higher activity consistently detected in the cell lysates compared with the supernatants at all-time points evaluated (*P* = 0.031). In addition, the variability (SD) was significantly lower in cell lysates than in supernatants (*P* = 0.031).

To confirm these observations, a wider range of viral inocula were used, with NA activity being measured 20 h post-infection. As shown in Fig 1B, NA activity was consistently higher in the lysates, with lower SDs, than in the supernatants, irrespective of the amount of virus used for infection. Next, NA-MNT was performed to compare NA activities between cell lysates and supernatants by testing sheep or human antisera against H5N1 vaccines. As shown in Fig 1C, The antibody titers based on the measurements of the NA activities in the cell lysates were slightly higher than those in the supernatants (three-fold higher), but no statistically significant differences were found when logarithmic titers were compared (*P*>0.05; Wilcoxon signed rank test). However, the variability observed in the supernatants was larger than that in the cell lysates, as revealed by a higher coefficient of variation (CV) for the antibody titers; specifically, the CV for the supernatants and lysates were 11.3–17.7% (mean, 13.7%) and 0–5.9% (mean, 3.4%), respectively.

### Reproducibility of the NA-MNT in comparison with the ELISA-MNT

To determine the reproducibility of NA-MNT, three individual samples were measured by NA-MNT in comparison with ELISA-MNT. Intra-assay precision was determined by conducting eight measurements on the same day while the inter-assay precision was determined by carrying out nine measurements from each sample over 3 days. The GMT values obtained from the two assays were found to be similar. As presented in Table 1, the mean intra-assay CV% in the NA-MNT was 0, which was lower than that in the ELISA-MNT. The inter-assay CV% range was 2.52–6.40 for NA-MNT and 4.92–11.06 for ELISA-MNT (Table 2). Although the titers of the tested samples were equal between the two assays, there was always less variability with the NA-MNT, revealing this assay has greater reproducibility.

**Table 1:** NA-MNT and ELISA-MNT intra-assay variation (n = 8).

Serum samples	NA-MNT			ELISA-MNT		
	Log_10_GMT	Log_10_SD	CV(%)	Log_10_GMT	Log_10_SD	CV(%)
**Low**	**1.60**	**0**	**0**	**1.64**	**0.11**	**6.49**
**Middle**	**2.51**	**0**	**0**	**2.54**	**0.11**	**4.19**
**High**	**4.01**	**0**	**0**	**4.05**	**0.11**	**2.63**

Intra-assay variations between the NA-MNT and ELISA-MNT were assessed using different titers of specific antisera (low, middle, and high reactive) against influenza H5N1 virus. GMT, geometric mean titer; SD, standard deviation; CV, coefficient of variation.

**Table 2:** NA-MNT and ELISA-MNT inter-assay variation (n = 9).

Serum samples	NA-MNT			ELISA-MNT		
	Log_10_GMT	Log_10_SD	CV(%)	Log_10_GMT	Log_10_SD	CV(%)
**Low**	**1.57**	**0.10**	**6.40**	**1.64**	**0.18**	**11.06**
**Middle**	**2.47**	**0.10**	**4.06**	**2.57**	**0.20**	**7.80**
**High**	**3.98**	**0.10**	**2.52**	**4.08**	**0.20**	**4.92**

Inter-assay variations between the NA-MNT and ELISA-MNT were assessed using different titers of specific antisera (low, middle, and high reactive) against influenza H5N1 virus. GMT, geometric mean titer; SD, standard deviation; CV, coefficient of variation.

### Correlations among the HI assay, ELISA-MNT, and NA-MNT for detection of anti-H5N1 antibodies

**Fig 2:**
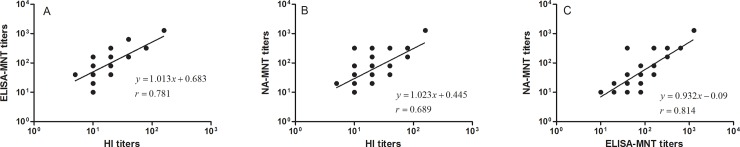
Correlations between the HI assay, ELISA-MNT, and NA-MNT for the measurements of anti-H5N1 antibodies. Antibodies were measured in 40 serum samples from healthy volunteers immunized with two doses of inactivated H5N1 influenza vaccine and compared between the (A) HI assay and ELISA-MNT (B) NA-MNT and HI assay, and (C) NA-MNT and ELISA-MNT. Linear regression equations and correlation coefficients were calculated by linear regression analysis of the log transformed data.

The correlations in the results obtained from the HI assay, ELISA-MNT, and NA-MNT were analyzed using serum samples collected from 40 healthy volunteers who received two doses of inactivated H5N1 influenza vaccine. All pre-immune sera tested negative for anti-H5N1 antibodies and the negative samples were assigned a value of 5 for subsequent calculations. For post-vaccination sera, lowest GMT values with the narrowest titer range were obtained using HI. Similar same range of titers was obtained with NA-MNT and ELISA-MNT, with the mean ratio of the logarithmic titers being 1.67 (Table 3). Seroconversion, defined as an antibody titer ≥1:40 after vaccination, was 75% by NA-MNT, slightly (but not significantly) lower than that of the ELISA-MNT (*P* = 0.152). In addition, both the ELISA-MNT and NA-MNT exhibited good correlations with the HI assay, with Spearman’s correlation coefficients being 0.781 (Fig 2A) and 0.689 (*P*<0.001), respectively (Fig 2B). However, a better correlation was found between ELISA-MNT and NA-MNT, with a Spearman’s correlation coefficient of 0.814 (*P*<0.001) (Fig 2C).

**Table 3:** Serology results from 40 healthy volunteers received two doses of inactivated H5N1 influenza vaccine.

Serum samples	GMT	Titer range	Number of subjects (%) with seroconversion
**HI**	**19**	**<10–160**	**11 (27.5)**
**ELISA-MNT**	**97**	**10–1280**	**35 (87.5)**
**NA-MNT**	**58**	**10–1280**	**30 (75.0)**

GMT, geometric mean titer.

### Correlations among the HI assay, ELISA-MNT, and NA-MNT for the detection of anti-H7N9 antibodies

**Fig 3:**
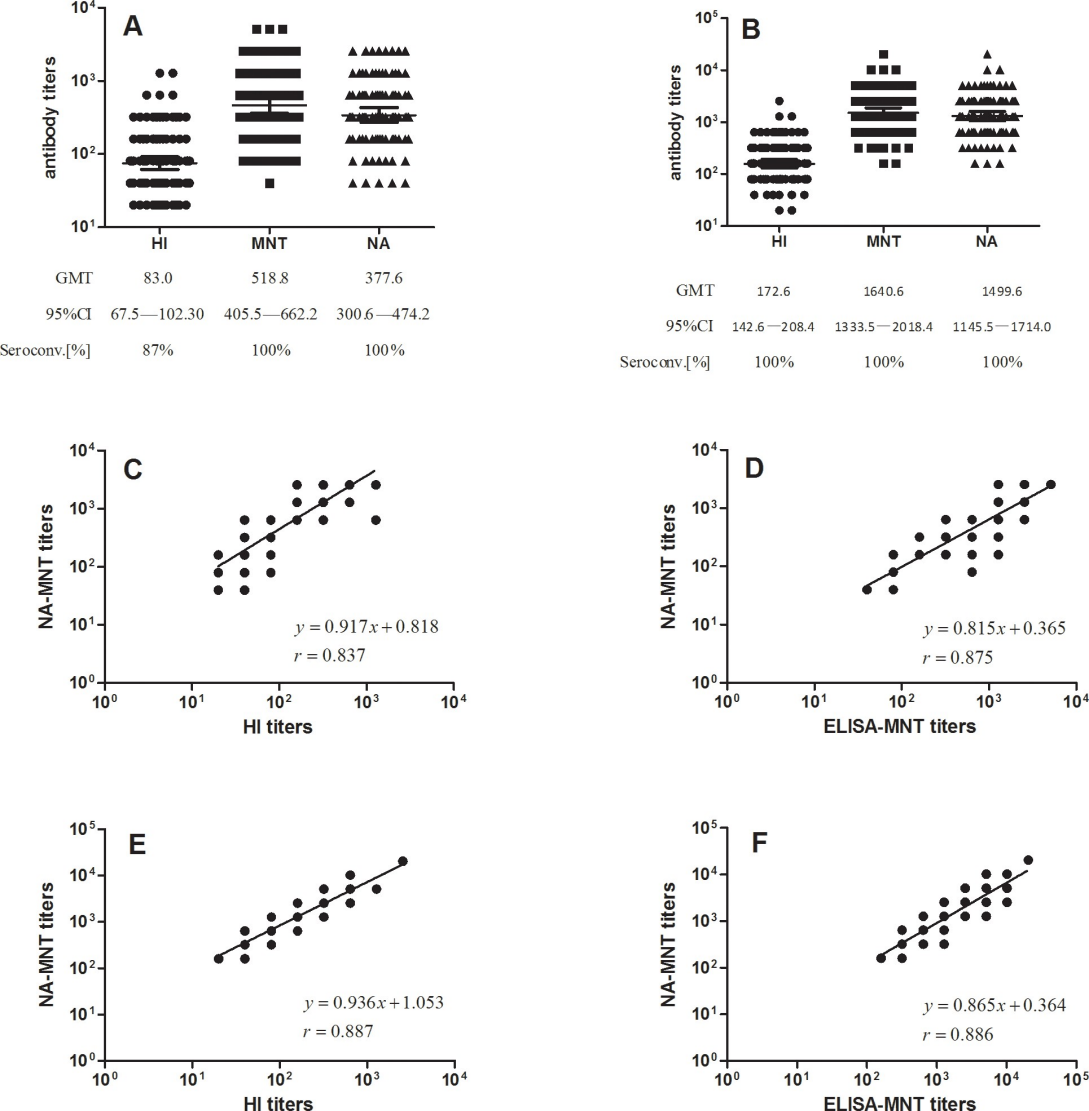
H7N9 antibody responses and correlations among the HI assay, ELISA-MNT, and NA-MNT. 92 serum samples from human subjects immunized with either (A) one dose or (B) two doses of candidate H7N9 influenza vaccine were analyzed using either HI assay, NA-MNT, or ELISA-MNT. The correlations among the titers in the 92 serum samples after immunization with one dose of candidate H7N9 influenza vaccines were measured by (C) HI assay versus NA-MNT or (D) ELISA-MNT versus NA-MNT. The correlations among the titers in the 92 serum samples after immunization with two doses of candidate H7N9 influenza vaccines were measured by (E) HI assay versus NA-MNT or (F) ELISA-MNT versus NA-MNT. Linear regression equations and correlation coefficients were calculated by linear regression analysis of the log transformed data.

We next investigated the correlations between the three assays for detection of anti-H7N9-specific antibodies. Serum samples from 92 human subjects received candidate H7N9 influenza vaccines were analyzed using the three assays. As observed with the H5N1 serum samples, all pre-immune sera were tested negative for H7N9 antibodies using all three assays. The antibody titers of serum samples harvested 21 days post-immunization were found to be slightly lower by NA-MNT than that by ELISA-MNT whereas significantly higher titers were found by NA-MNT than HI assay. As shown in Fig 3A, the seroconversion rates in the NA-MNT and ELISA-MNT were 100%, which was higher than that in the HI assay (87%).

For serum samples harvested 42 days after immunization, higher GMT values were obtained by both ELISA-MNT and NA-MNT compared to that by the HI assay (Fig 3B). Linear regression analysis was then performed to assess the correlation among the assays. There was good correlation between the NA-MNT and HI assay for sera obtained on day 21 (*r* = 0.837, *P*<0.01) (Fig 3C), similar to that for sera from subjects who received two vaccinations (*r* = 0.887, *P*<0.01) (Fig 3E). Moreover, there was a good correlation between the NA-MNT and ELISA-MNT in the measurement of anti-H7N9 antibodies from subjects who received either one or two doses of the vaccine. As shown in Fig 3D and 3F, the Spearman’s correlation coefficient was 0.875 or 0.866 for individuals receiving one or two immunizations, respectively. Taken together, these data indicate strong correlations among the results obtained from the three assays.

## Discussion

Quantitation of influenza neutralization antibodies is important for influenza epidemiological studies and vaccine evaluations [4, 7, 12]. Because the detection of CPEs induced by influenza virus is quite laborious and subjective, NP is detected by ELISA to analyze neutralizing antibodies in cell cultures [13, 14]. However, ELISAs involve multiple steps such as cell fixation, antibody incubation, washing, and color development, each of which may contribute to high intra-laboratory variation as reported in an international study [8, 15]. In addition, the quality and the source of the reagents, such as the antibody used for NP capture, likely affect the test results. Therefore, a simple and reliable assay might facilitate the development of standardized assays for vaccine evaluation.

In this report, we present a novel neutralization assay that employs NA activity as an indicator of viral replication. NA is a major glycoprotein on the surface of influenza virus. It plays an important role in viral infection by promoting the release of influenza virus from infected cells and facilitating viral infection to adjacent uninfected cells. A fluorometric method using 4-MU-NANA as a substrate for NA activity tests was first described in 1979, and this method was found not to be affected by cell culture media [16]. The FL-MU-NANA assay was initially developed to evaluate influenza virus replication in cells, since the NA activity of live influenza viruses is correlated with the amount of virus [17]. Therefore, NA activity could be used as readout for the neutralization test, enabling simpler and faster virus detection, given that the only step is to add 4-MU-NANA to the cell supernatant or lysate after incubation with virus and serum mixtures.

Unlike the ELISA-MNT, in which the nucleoprotein is quantified, the NA activity of influenza virus particles can be measured in either culture supernatants or infected cells. In the current study, we found that NA activity was higher in cell lysates than supernatants, irrespective of the amount of viral inoculum and incubation time. This is likely because most viruses remained in the cells after the 20 h incubation, which is close to one viral replication cycle. Moreover, NA-MNT using cell lysates demonstrated less variability, with intra- and inter-assay CVs being less than 10%. While it remains unclear as to why the results from the modified NA-MNT were less variable, higher NA activities in cell lysates may have contributed to the improved reproducibility.

The current study showed that the NA-MNT results were highly correlated with the ELISA-MNT results when measuring avian influenza vaccine-induced antibodies. The titers obtained by NA-MNT in both H5N1 and H7N9 sera were slightly lower than those obtained by ELISA-MNT. However, this difference is acceptable since the ratios of the logarithmic titers between the two assays were less than two-fold (one more sample dilution). This observation is not completely surprising since both assays are cell-based MNTs but with different readout. However, higher serum titers were found with both MNTs than with the HI assay, which is consistent with other studies reporting that MNTs are more sensitive than HI assays in detecting the antibody response to seasonal and avian influenza [13, 14, 18]. MNTs measure functional antibodies targeting hemagglutinin and other envelope glycoproteins, whereas the HI assay detects responses to the HA component only. A study of influenza neutralization assays that used viral NA activity to quantify influenza replication (AVIAN assay) found that pre- and post vaccination titers were higher in the AVIAN assay than in the ELISA-MNT [19]. However, our results demonstrated that all pre-immune serum samples tested negative (<10) for anti-H5N1 and -H7N9 antibodies. In addition, there were no significant differences in the post-vaccination titer, titer range, or seroconversion rate between the two cell-based neutralization assays. This might be due to differences in the method used to measure enzymatic activity indicative of viral replication and the characteristics of the serum antibodies, which targeted pandemic but not seasonal influenza.

Although ELISA-MNT is known to be a sensitive and rapid method for vaccine evaluations and sero-epidemiological studies, it is associated with some drawbacks. Specifically, the acetone fixation step may cause detachment of partial cells, which reduced the specific signal from infected cells and elevated the background values to the space left on the plate. This may result in significant variation and even yield false-positive results. In contrast, no fixation step is employed in the NA-MNT described here, with direct lysis of cells by Tween 20-containing buffer. For detection of downstream NA activity, a substrate is added directly to the lysed cells for detection. The relatively simpler methods used in this assay not only shorten the test period but also increase the robustness of the assay. Indeed, the NA-MNT has demonstrated reduced intra- and inter-laboratory variability with the ELISA-MNT. It is of note that the objective of this study was to analyze antibody responses to influenza H5 and H7 vaccines using the NA-MNT, so we did not assess the applicability of the NA-MNT to the detection of other influenza viruses, especially influenza viruses with lower NA activity. In future studies, more virus strains would be included to determine a wider application of this method.

In conclusion, the current results demonstrated that the NA-MNT is a rapid and reliable method that could be easily performed to evaluate neutralizing antibodies against candidate pandemic influenza vaccines.

## Supporting information

S1 Table Raw data of Table 1 NA-MNT and ELISA-MNT intra-assay variations. (DOCX)

S2 Table Raw data of Table 2 for NA-MNT and ELISA-MNT inter-assay variations. (DOCX)

S3 Table Raw data for Table 3 Serology results from 40 healthy volunteers who received two doses of the inactivated H5N1 influenza vaccine. (DOCX)

S4 Table Raw data of Fig 1A Comparison of NA activity between cell lysates and supernatants. (DOCX)

S5 Table Raw data of Fig 1C Comparison of NA activity between cell lysates and supernatants. (DOCX)
