# Supplementary material for: A neuraminidase activity-based microneutralization assay for evaluating antibody responses to influenza H5 and H7 vaccines
Source: PLoS One. 2018 Nov 15;13(11):e0207431. doi: 10.1371/journal.pone.0207431 (PMC6237356; doi:10.1371/journal.pone.0207431)
Supplement: S1 Table — (DOCX) [file pone.0207431.s001.docx]

**S1 Table. Raw data of Table 1 NA-MNT and ELISA-MNT intra-assay variations**

| **Serum**  **samples** | NA-MNT titer | | |  | ELISA-MNT titer | | |
| --- | --- | --- | --- | --- | --- | --- | --- |
|  | **Low** | **Middle** | **High** |  | **Low** | **Middle** | **High** |
| 1 | 40 | 320 | 10240 |  | 40 | 320 | 10240 |
| 2 | 40 | 320 | 10240 |  | 40 | 320 | 10240 |
| 3 | 40 | 320 | 10240 |  | 80 | 320 | 10240 |
| **4** | 40 | 320 | 10240 |  | 40 | 320 | 10240 |
| **5** | 40 | 320 | 10240 |  | 40 | 320 | 10240 |
| **6** | 40 | 320 | 10240 |  | 40 | 320 | 10240 |
| **7** | 40 | 320 | 10240 |  | 40 | 640 | 10240 |
| **8** | 40 | 320 | 10240 |  | 40 | 320 | 20480 |

Note: Intra-assay variations of the NA –MNT and ELISA-MNT assay were assessed using different titers of specific antisera (low ,middle and high reactive) against influenza H5N1 virus.
