# Supplementary material for: A neuraminidase activity-based microneutralization assay for evaluating antibody responses to influenza H5 and H7 vaccines
Source: PLoS One. 2018 Nov 15;13(11):e0207431. doi: 10.1371/journal.pone.0207431 (PMC6237356; doi:10.1371/journal.pone.0207431)
Supplement: S3 Table — (DOCX) [file pone.0207431.s003.docx]

**S3Table.Raw data for Table 3 Serology results from 40 healthy volunteers who received two doses of the inactivated H5N1 influenza vaccine**

| **Serum**  **sample** | Post-vaccination titer | | | **Serum**  **sample** | Post-vaccination titer | | |
| --- | --- | --- | --- | --- | --- | --- | --- |
|  | **HI** | **ELISA-**  **MNT** | **NA-**  **MNT** |  | **HI** | **ELISA-**  **MNT** | **NA-**  **MNT** |
| 1 | 10 | 40 | 20 | **21** | 20 | 160 | 80 |
| 2 | 20 | 160 | 80 | **22** | 40 | 160 | 40 |
| 3 | 40 | 160 | 80 | **23** | 10 | 20 | 20 |
| **4** | 40 | 160 | 80 | **24** | 20 | 160 | 80 |
| **5** | 5 | 40 | 20 | **25** | 20 | 160 | 320 |
| **6** | 10 | 40 | 20 | **26** | 10 | 40 | 10 |
| **7** | 5 | 40 | 20 | **27** | 10 | 10 | 10 |
| **8** | 10 | 40 | 40 | **28** | 20 | 40 | 320 |
| **9** | 20 | 80 | 80 | **29** | 10 | 20 | 20 |
| **10** | 20 | 80 | 80 | **30** | 10 | 80 | 40 |
| **11** | 40 | 160 | 80 | **31** | 20 | 320 | 160 |
| **12** | 10 | 20 | 10 | **32** | 10 | 80 | 40 |
| **13** | 10 | 20 | 10 | **33** | 80 | 320 | 160 |
| **14** | 10 | 80 | 10 | **34** | 40 | 640 | 320 |
| **15** | 20 | 80 | 40 | **35** | 160 | 1280 | 1280 |
| **16** | 20 | 80 | 20 | **36** | 10 | 160 | 320 |
| **17** | 20 | 160 | 80 | **37** | 20 | 80 | 40 |
| **18** | 40 | 160 | 80 | **38** | 80 | 320 | 320 |
| **19** | 20 | 160 | 80 | **39** | 80 | 320 | 160 |
| **20** | 10 | 160 | 80 | **40** | 40 | 160 | 40 |

Note: Pre-vaccination titers were all negative detected by HI,ELISA-MNT or NA-MNT assay , the sample titer was <10.,So the detailed data was omitted.
