# Supplementary material for: A neuraminidase activity-based microneutralization assay for evaluating antibody responses to influenza H5 and H7 vaccines
Source: PLoS One. 2018 Nov 15;13(11):e0207431. doi: 10.1371/journal.pone.0207431 (PMC6237356; doi:10.1371/journal.pone.0207431)
Supplement: S4 Table — (DOCX) [file pone.0207431.s004.docx]

**S4 Table 4. Raw data of Fig 1A Comparison of NA activity between cell lysates and supernatants**

| **time**  **point（h）** | cell lysates(RFU) | | |  | Supernatant(RFU) | | |
| --- | --- | --- | --- | --- | --- | --- | --- |
|  | **1** | **2** | **3** |  | **1** | **2** | **3** |
| 0 | 25.77 | 21.92 | 22.99 |  | 21.534 | 14.143 | 18.45 |
| 6 | 27.42 | 25.06 | 24.36 |  | 13.485 | 29.780 | 18.07 |
| 12 | 392.09 | 397.25 | 402.02 |  | 128.775 | 89.250 | 168.64 |
| 18 | 692.09 | 673.85 | 687.72 |  | 351.785 | 420.950 | 257.34 |
| 24 | 900.49 | 905.55 | 925.42 |  | 552.350 | 648.750 | 740.26 |
| 36 | 1534.27 | 1504.66 | 1515.23 |  | 1035.895 | 1297.750 | 1324.65 |

Note: 1,2,3 represent three different experiment
