# Supplementary material for: A neuraminidase activity-based microneutralization assay for evaluating antibody responses to influenza H5 and H7 vaccines
Source: PLoS One. 2018 Nov 15;13(11):e0207431. doi: 10.1371/journal.pone.0207431 (PMC6237356; doi:10.1371/journal.pone.0207431)
Supplement: S5 Table — (DOCX) [file pone.0207431.s005.docx]

**S5 Table 5. Raw data of Fig 1C Comparison of NA activity between cell lysates and supernatants**

| [number](file:///C:\Program%20Files%20(x86)\Youdao\Dict\7.5.2.0\resultui\dict\?keyword=number)  [of](file:///C:\Program%20Files%20(x86)\Youdao\Dict\7.5.2.0\resultui\dict\?keyword=of)  [experiment](file:///C:\Program%20Files%20(x86)\Youdao\Dict\7.5.2.0\resultui\dict\?keyword=experiment) | cell lysates(antibody titer) | | | |  | Supernatant(antibody titer) | | | |
| --- | --- | --- | --- | --- | --- | --- | --- | --- | --- |
|  | shRef  H5N1 | HuHiPos | HuMoPos | HuNeg |  | shRef  H5N1 | HuHiPos | HuMoPos | HuNeg |
| 1 | 5120 | 640 | 160 | 5 |  | 2560 | 160 | 80 | 5 |
| 2 | 10240 | 640 | 160 | 5 |  | 10240 | 640 | 40 | 10 |
| 3 | 10240 | 640 | 160 | 5 |  | 1280 | 320 | 160 | 5 |
| 4 | 10240 | 640 | 320 | 5 |  | 1280 | 160 | 40 | 5 |
| 5 | 10240 | 320 | 160 | 5 |  | 5120 | 640 | 80 | 5 |

Note: HuNeg antibody titers were detected as <10, assigned a titer of 5 for calculation
